# Polyphenolic Profile and Biological Activity of Chinese Hawthorn (*Crataegus pinnatifida* BUNGE) Fruits

**DOI:** 10.3390/molecules171214490

**Published:** 2012-12-06

**Authors:** Tunde Jurikova, Jiri Sochor, Otakar Rop, Jiri Mlcek, Stefan Balla, Ladislav Szekeres, Vojtech Adam, Rene Kizek

**Affiliations:** 1 Department of Natural and Informatics Sciences, Faculty of Central European Studies, Constantine the Philosopher University in Nitra, Drazovska 4, SK-949 74 Nitra, Slovak Republic; E-Mails: tjurikova@ukf.sk (T.J.); lszekeres@ukf.sk (L.S.); 2 Vysoka skola Karla Englise, Sujanovo nam. 356/1, CZ-602 00, Brno, Czech Republic; E-Mail: sochor.jirik@seznam.cz; 3 Department of Food Technology and Microbiology, Faculty of Technology, Tomas Bata University in Zlin, Namesti T. G. Masaryka 275, CZ-762 72 Zlin, Czech Republic; E-Mails: rop@ft.utb.cz (O.R.); mlcek@ft.utb.cz (J.M.); 4 Department of Chemistry and Biochemistry, Mendel University in Brno, Zemedelska 1, CZ-613 00 Brno, Czech Republic; E-Mail: vojtech.adam@mendelu.cz; 5 Central European Institute of Technology, Brno University of Technology, Technicka 3058/10, CZ-616 00 Brno, Czech Republic; E-Mail: kizek@sci.muni.cz

**Keywords:** *Crataegus pinnatifida*, polyphenolic compounds, antioxidant activity, biological value of fruit

## Abstract

Chinese hawthorn (*Crataegus pinnatifida* Bge.) fruits are rich in polyphenols (e.g., epicatechin, procyanidin B2, procyanidin B5, procyanidin C1, hyperoside, isoquercitrin and chlorogenic acid)—active compounds that exert beneficial effects. This review summarizes all information available on polyphenolic content and methods for their quantification in Chinese hawthorn berries and the relationships between individual polyphenolic compounds as well. The influence of species or cultivars, the locality of cultivation, the stage of maturity, and extract preparation conditions on the polyphenolic content were discussed as well. Currently, only fruits of *C. pinnatifida* and *C. pinnatifida* var. *major* are included in the Chinese Pharmacopoeia. Recent trials have demonstrated the efficacy of Chinese hawthorn fruit in lowering blood cholesterol and the risk of cardiovascular diseases. The fruit has also demonstrated anti-inflammatory and anti-tumour activities. This review deals mainly with the biological activity of the fruit related to its antioxidant properties.

## 1. Introduction

The medicinal properties of hawthorn (*Crataegus* spp.—the genus comprises approximately 300 species) have been utilized by many cultures since antiquity for a variety of therapeutic applications [1,2]. Various species of hawthorn have been widely using as medicinal materials and foodstuffs in China and the European countries, among which *Crataegus monogyna* and *Crataegus laevigata* are the major hawthorn species used in Europe, and *Crataegus pinnatifida* and *Crataegus scabrifolia* in China and Asian countries [3,4,5,6].

The Chinese hawthorn is commonly considered to comprise 18 species, of which *Crateagus pinnatifida* Bge. and its “Shanlihong” (*C. pinnatifida* Bge. var. *major* N.E.Br.) variety are the most important, due to their large and delicious fruits with a characteristic acidic taste [5,7]. Moreover, currently, only fruits of *C. pinnatifida* and *C. pinnatifida* var. *major* are included in the Chinese Pharmacopoeia [8]. The fruits of *Crateagus pinnatifida* and *Crataegus scabrifolia* have been used traditionally as a peptic agent in oriental medicine and recently in a local soft drink product [9], in jams, juices and tinned foods, and as a basic ingredient for making wines and various sweet foods [10].

Hawthorn species (*Crataegus* spp.) have recently attracted increasing attention in the field of food nutraceuticals and medicine because of their widely reported health benefits. Recent trials have demonstrated their efficacy in lowering blood cholesterol and in the reduction of the risk of cardiovascular diseases [7,11,12,13], which can be considered as the most significant among other lesser known fruit species [14,15,16]. Nowadays it is also well known that all these mentioned beneficial health promoting activities are connected with polyphenolic compounds and triterpene acids [8]. Numerous reviews on the chemical composition of *Crateagus* spp. along with their health promoting activity have been published. This review focuses its attention only in one species—Chinese hawthorn (*Crateagus pinnatifida* Bge.) and summarises its health benefits.

## 2. Polyphenolic Profile of Chinese Hawthorn Fruits

The Chinese hawthorn fruit has been shown to have high polyphenolic content [17], with clear synergistic effects between the different phenolic compounds [18]. Generally, the total content of phenolics in *Crataegus* sp. fruit was 3.54% expressed as gallic acid equivalents [19]. Total content of polyphenols in Chinese hawthorn fruit *C. pinnatifida* Bge. was 96.9 ± 4.3 mg gallic acid equivalents per gram weight, measured by the Folin-Ciocalteu reagent method [7].

In fruits, oligomeric procyanidins and their glycosides represent the major group of phenolic compounds [8,20]. Investigations on the phenolic profile in fruits of the European hawthorn species *C. monogyna* have shown the presence of flavonol glycosides, phenolic acids and B-type procyanidins [20,21,22,23]. Phenolics, mainly flavonoids and proacyanidins belonging to the B-type procyanidins (PAs) class are also considered among the most important bioactive compounds in Chinese hawthorn *C. pinnatifida* Bge. fruits [7,24,25]. The rest are flavonol glycosides, anthocyanins or phenolic acids [7]. Among more than 40 phenolic compounds in Chinese hawthorn fruits (*C. pinnatifida* Bge.var. *major* N.E.Br.), the following can be considered as the main constituents: procyanidin B2, epicatechin, chlorogenic acid, procyanidin C1 and rutin [7]. According to PCA plot analyses varieties of *C. pinnatifida* var. *major* can be divided into two groups, one rich in procyanidins and acids and another rich in flavonoids and sugars [7].

### 2.1. Phenolic Acids

**Figure 1:**
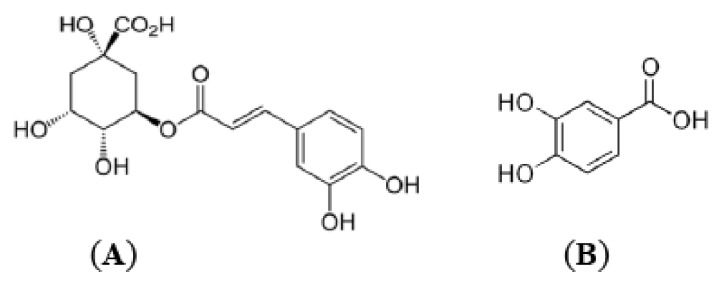
The most frequent phenolic acids—(**A**) chlorogenic acid; (**B**) protocatechuic acid, in Chinese hawthorn fruits.

Chlorogenic acid (**A**, Figure 1) has been found in fruits and leaves of all hawthorn species investigated [8]. The presence of this compound in fruit of Chinese hawthorn was confirmed by several studies [7,14,17,25]. However, its isomer 5-*O*-caffeoylquinic acid (neochlorogenic acid) was reported only in fruits of *C. grayana* [8,25]. In addition to the dominant phenolic acid in Chinese hawthorn fruit, 4-hydroxybenzoic acid [26], 4-aminohydroxybenzoic acid along with gallic acid [14,27] were also present. The presence of protocatechuic acid (**B**, Figure 1) in fruit of *C. pinnatifida*. Bge. caffeic acid was only seen in fruit of European hawthorn (*C. monogyna*) [28].

### 2.2. Flavonoids

To date, more than 50 flavonoids have been isolated from genus *Crateagus* spp. [8,29,30]. Determination of total flavonoids aglycone content gave 0.18%. The percentage of hyperoside, as the main flavonol component, was 0.14% [19]. Aside from hyperoside [31], the presence of *O*-glycosides like luteolin-7-glycoside, rutin, and the *C*-glycosides vitexin, vitexin rhamnoside and monoacetylvitexin rhamnoside in *Crateagus* sp. fruits was also confirmed. Regarding the total contents of flavonol glycosides, the fruits of *C. pinnatifida* var. *major* contained less flavonol glycosides (0.4 mg/G DM as average content of 10 cultivars in the species) than those of other species. On the other hand, the fruit of *C. pinnatifida* var. *major* contained the highest level (1.1 mg/g DM) among all the tested hawthorn species [25].

**Figure 2:**
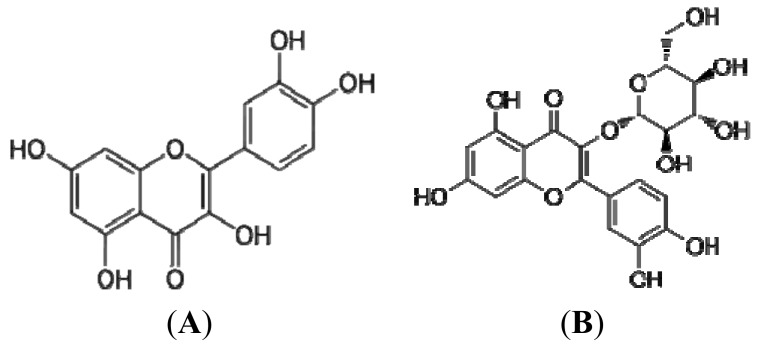
The predominant flavonoids—(**A**) quercetin and (**B**) isoquercetin in Chinese hawthorn fruits.

Kaempferol is the dominant flavonol in Chinese hawthorn fruit [7], however, the presence of rutin in some cultivars of *C. pinnatifida* var. *major* was confirmed [7,25]. Compounds of the same molecular weight were found in fruits of *C. pinnatifida* var. *major* and were identified as quercetin rhamnosyl hexoside and quercetin (dirhamnosyl hexoside) and isoquercetin [25] (Figure 2). Most *C*-glycosyl flavones in hawthorn berries are derivatives of apigein and luteolin [8]. Cui *et al*. [10] determined the polyphenol profile of four types of Chinese hawthorn fruit extract by high performance liquid chromatography with UV detection (HPLC-UV) profile and found vitexin-2''-*O*-rhamnoside—one of the typical components of Chinese hawthorn leaves.

### 2.3. Procyanidins as the Main Component of Chinese Hawthorn Fruit

Procyanidins, as the second most abundant group of natural phenolics after lignins, are widespread throughout the plant kingdom where they display multiple biochemical properties, mainly involving interactions with proteins, the chelation of metals and antioxidant activity, which are the basis of their various protective functions for plants [32,33,34].

**Figure 3:**
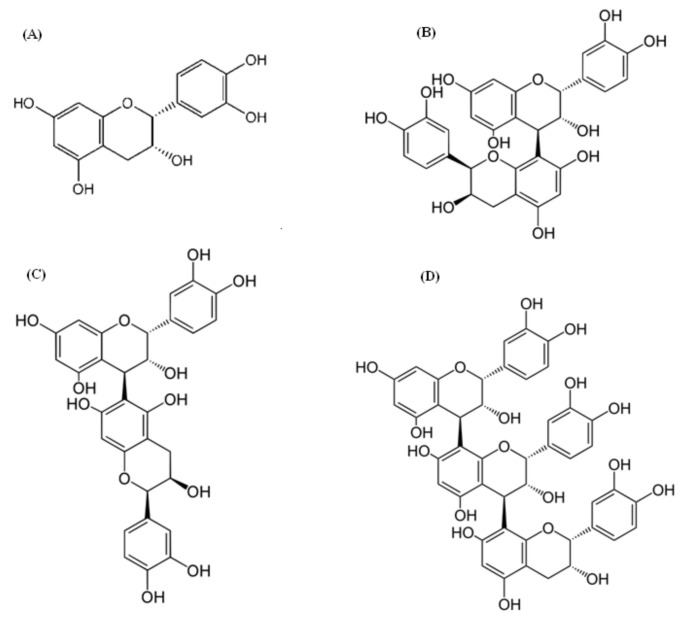
The most frequent procyanidin monomer (**A**) epicatechin dimers procyanidin B2 (**B**) and procyanidin B5 (**C**) and trimer procyanidin C1 (**D**) in Chinese hawthorn fruits.

Procyanidins are a class of proanthocyanidins consisting primarily of epicatechin as the flavan-3-ol units or catechin. Epicatechin (**A**, Figure 3) predominates in fruits of Chinese hawthorn. The content of epicatechin in the fruits analysed varied from 0.9–11.7 mg/g DM. Most samples contained between 2 and 6 mg/g DM of epicatechin, but several samples had extremely high levels of the compound, especially “Shandongdajinxing” of *C. pinnatifida* var. *major* (11.7 mg/g DM) [7,25]. Svedstrom *et al*. also identified catechin in fruits of *Crataegus* spp. in monomer form and as a constituent unit of oligomeric and polymeric procyanidins [23]. On the other hand, catechin has not been identified in Chinese hawthorn fruits [10,25]. For procyanidins of each degree of polymerization, several isomers may exist. In Chinese hawthorn fruits, the yields of procyanidins monomer, dimer, trimer, tetramer and pentamer were 50.5%, 30.3%, 23%, 14.6% and 12.5%, respectively [17].

The diversity of procyanidins is mainly due to their compositional differences and sequential order of the flavan-3-ol units, as well as variation in the location and stereochemistry (A/B) of the interflavanol bonds. The number of possible isomers increases exponentially with the degree of polymerization. The isomers have the same molecular weight and similar UV and mass spectral characteristics [8]. Liu *et al*. identified procyanidins in Chinese hawthorn fruits, based on UV and mass spectra, that were mostly B-type procyanidins (PA) and their glycosides, including aglycons of three dimers, three trimers, eight tetramers, four pentamers, two hexamers and two glycosides of PA monomers, seven glycosides of PA dimers, one glycoside of a PA trimer, two glycosides of PA tetramers, one glycoside of a PA pentamer, and two glycosides of quercetin [25]. According to [7] epicatechin, PA dimers, PA dimer hexosides and PA trimers were the major components, representing 64% of the total procyanidin peak area [7].

Procyanidins B2 and B5 (**B** and **C**, Figure 3) were identified as the major procyanidin dimers and procyanidin C1 (**D**, Figure 3) as the major procyanidins (PC) trimer in fruits of *C. pinnatifida* var. *major* [8,25]. The content of procyanidin monomers (epicatechin) and dimers (procyanidins B2 and B5 were 0.78%), procyanidin trimers (C1, 2, 3) made up 0.40%, or approximately half of the content of monomers and dimers. The degree of polymerization in Chinese hawthorn fruit was reduced to 1.39 (polyphenolic extract) to 1.66 (in ethyl acetate extract) [10]. Values of procyanidins in Chinese hawthorn berries are lower in comparison with European hawthorn fruit [23] which might be related to higher level of organic acids seen in Chinese hawthorn fruit [17] and the decomposition of procyanidin compounds into smaller fragments under acidic conditions [7]. *C. scabrifolia* Rehd., and *C. brettschneideri* Ahneid. have similar procyanidin aglycon profiles, whereas the profile of procyanidin glycosides varied among the mentioned species. Fruits of *C. pinnatifida* var. *major* had higher contents of procyanidins, but lower contents of flavonols compared with *C. brettschneideri*. The fruits of *C. scabrifolia* contained the highest level of PA dimer hexoside, which was detected in trace amounts in the fruit of *C. pinnatifida* Bge. The content of this compound in fruit of different cultivars of *C. pinnatifida* var. *major* varied from trace amounts to 0.8 g/kg DM [25].

### 2.4. Methods for Quantification of Polyphenolic Compounds in Chinese Hawthorn Fruits

The first attempts of quantification of total polyphenols content in *Crateagu*s sp. fruits were based on the colorimetric Folin-Ciocalteu method. Froehlicher *et al*. found out that dried and fresh fruit of *C. monogyna* contained 12 g gallic acid equivalent/kg DM [28]. Content of procyanidins was determined in fruit of Chinese hawthorn by a gravimetric method after acetone/water extraction and acidic precipitation. *C. pinnatifida*, *C. pinnatifida* var. *major* contained 18 and 61 g/kg DM [35]. The mentioned methods were used only for rough estimation of polyphenol content in hawthorn berries [8]. Nowadays, many methods, including capillary zone electrophoresis with electrochemical detection, HPLC with electrochemical detection (ED) and HPLC with mass spectrometry (MS) have been reported for determination and quantification of polyphenolic compounds in fruits [15].

Other approaches have utilized the HPLC-UV technique. Cui *et al*. quantified the levels of seven polyphenols (epicatechin, procyanidin B2, procyanidin B5, procyanidin C1, hyperoside, isoquercitrin and chlorogenic acid) in mature fruits of Chinese hawthorn (*C. pinnatifida*) by a HPLC-UV method [17]. The average contents of those constituents in 37 representative cultivars were 1,405, 1,505, 339, 684, 56, 41, 234 μg/g FW, respectively. The content of two dominant flavonoids was equivalent to 1/40 of four major procyanidin components, indicating that procyanidins are the major active constituents of Chinese hawthorn fruits [10].

Urbonaviciute *et al*. utilized capillary electrophoretic analysis of flavonoids in hawthorn ethanolic extracts of fruits [20]. Liu *et al*. isolated by this method four flavones from *C. pinnatifida* with the following recoveries: vitexin-2'-rhamnoside 96.8%, hyperoside 99.9%, rutin 97.1% and vitexin 97.8% [36]. The content of the mentioned flavones was higher in leaves than in fruits, and hyperoside was not detected in either *C. pinnatifida* fruits or flowers. A method based on capillary electrophoresis with electrochemical detection was also developed for the simultaneous separation and determination of epicatechin, kaempferol, chlorogenic acid and 4-hydroxybenzoic acid [26]. Quercetin and protocatechuic acid was detected in Chinese hawthorn fruit by this method for the first time.

Nowadays an electrochemical detection method for polyphenolic compounds has been widely utilized in analyses of fruit samples of common and less known fruit species as well [37,38,39]. HPLC coupled with electrochemical detection was also performed on samples of less common fruit genotypes, including Chinese hawthorn, blue honeysuckles and Saskatoon fruit [40]. Among the fruits belonging to the *Rosaceae*, Hawthorn fruit (*C. pinnatifida* Bge.), showed a highest rate of neuroprotective phenols (gallic acid, 4-aminobenzoic acid, rutin and quercitrin) with the grand total of 437 mg/100g FW. The fruits of *C. pinnatifida* had almost four times higher neuroprotective phenolics content compared to the plant species *Lonicera edulis* Turcz. ex. Freyn and *Amelenchier canadensis* investigated by Gazdik *et al*. [14]. This can account for the considerable antioxidant activity of this plant species. Among analysed neuroprotective phenolics the concentration of 4-aminobenzoic acid dominated (970 mg/kg FW). This observation is consistent with the higher phenolic content of Chinese hawthorn fruit with respect to the other fruits described [41,42,43,44,45].

Liu *et al*. provided the first systematic study of phenolic compounds in the major Chinese hawthorn variety *C. pinnatifida* Bge. var. *major* [7,25]. The phenolics were extracted from the fruits with 80% aqueous ethanol. In order to improve the separation between compounds, especially between procyanidins, the extract was further fractionated by polyamide column chromatography, followed by analyses of each fraction by HPLC equipped with a diode array detection system and HPLC-MS. According to the results the mentioned authors identified the presence of ideain (cyanidin-3-*O*-galactoside), chlorogenic acid (0.2–1.6 mg/g DM) as the main phenolic acid, flavonol glycosides—hyperoside (quercetin-3-*O*-galactoside) (0.1–0.8 mg/g dry mass DM), and isoquercitrin (quercetin-3-*O*-glucoside) (0.1–0.3 mg/g DM), which is in good agreement with the previously published results [23,28]. The level of chlorogenic acid in samples of *C. pinnatifida* was higher than had been reported previously in 37 cultivars of *C. pinnatifida* var. *major* [10]. Procyanidins were eluted from the polyamide column in a sequence related to degree of polymerisation, and the glycosides eluted earlier than the corresponding aglycons. The total content of procyanidins in fruit of *C. pinnatifida* var. *major* reported by [7] was comparable to the level (23 g/kg DM) reported by [17], with slight deviations possibly due to the different cultivars examined in the two studies [8]. Liu *et al*. [7] developed a simple and very specific high-performance liquid chromatographic method for pharmacokinetics study of hyperoside isolated from hawthorn in rat plasma after intravenous administration and they results indicated that pharmacokinetics in rats obeyed nonlinear process. Among procyanidins, monomers of A-type and B-type hexamers were identified, with predominance of procyanidin B2 (epicatechin-(4β→8)-epicatechin) and epicatechin (0.9–11.7 mg/g DM). PA B2 (0.7–12.4 mg/g DM), PA dimer II (0.1–1.5 mg/g DM), PA trimer I (0.1–2.7 mg/g DM), PA trimer II (0.7–6.9 mg/g DM), PA trimer III (0.01–1.2 mg/g DM) and a PA dimer hexoside (trace–1.1 mg/g DM).

Cheng *et al*. developed a simultaneous determination of vitexin-2''-*O*-glucoside and vitexin-2''-*O*-rhamnoside (VOR), rutin and hyperoside in the extract of *Crateagus pinnatifida* Bge. leaves and fruits [46]. Another novel system for simultaneous determination of vitexin, vitexin-2''-O-rhamnoside, rutin and hyperoside in the ethanol extract from hawthorn fruits is microemulsion liquid chromatography (MELC) which gives mean recoveries in the 98.6–101.6% range [47]. In another study [25], phenolic compounds in extracts of 22 hawthorn samples belonging to four species/varieties were determined, with the most abundant glycosides being hyperoside and isoquercetin.

Ying *et al*. developed a HPLC method with high recovery (95.4 ± 1.3%) for the investigation of volatile organic compounds (VOR) by determining malondialdehyde (MDA) in ECV304 cell culture medium [48]. A novel ionic liquid-based pressurized liquid extraction (IL-PLE) procedure coupled with high performance liquid chromatography (HPLC) tandem chemiluminescence (CL) detection capable of quantifying trace amounts of rutin and quercetin in *C. pinnatifida* Bunge, was described [49]. Under the optimized conditions, good reproducibility of the extraction was obtained and good linearity was observed, with correlation coefficients (*r*) between 0.9997 and 0.9999.

### 2.5. Stability of Polyphenolic Compounds in Chinese Hawthorn Fruits

The phenolic composition in hawthorn fruit varies among species and cultivars [25,35] accounted for 80% of variance between species of *C. pinnatifida* and *C. brettschneideri*. Geographic locations also had to be considered. Significant inverse correlation between procyanidin contents and the latitude of the geographical origin of the studied cultivars was observed (*r* = 0.3851, *p* < 0.02). The time of harvest is also a very important factor [13]. The total phenolic content was influenced by stage of maturity, thus in *C. pinnatifida* Hebei Dajinxing cv. 61 days after blossom, the total polyphenol levels reached their highest point at 1.36 g/100 g FW. Fruit shows changes in levels of procyanidins, flavonoids and chlorogenic acid. In the early bearing period, the chlorogenic acid and flavonoid levels were relatively higher. In the fruit of *C. pinnatifida* var. *major*, procyanidins were synthesised rapidly in the early growth stage and reached up to 14 g/kg FW in fruits, thereafter the procyanidin level in fruit decreased gradually, reaching 4 g/kg FW at the optimal maturity stage [10]. Similarly, in fruits of *C. grayana* the content of phenolic compounds were the highest around mid-August and early September (22 g/kg DM) [25]. Phenolic compounds in hawthorn fruit are reported to be pH sensitive, with high stability being seen under acidic conditions [27]. The values are natural because of the high content of organic acids (3–6% caffeic, malic, tartaric and citric acids in dried fruit). Moreover, Chinese hawthorn fruit has higher acid content in comparison with popular fruits of European hawthorn [17]. Cui *et al*. developed a new extraction procedure that uses ethyl acetate treatment of an ethanol extract to prepare a sugarless extract [10]. They found that carbohydrates reduced the concentration of polyphenols and hastened procyanidin decomposition in an environment of moist acidity, therefore, the yield of polyphenols is also influenced by the extraction conditions used. According to Vierling *et al*. [50], procyanidins, as the main components of hawthorn fruit, can be easily extracted by 70% ethanol. Cui *et al*. found out that procyanidins eluted faster in chromatograms when a low ratio of acetonitrile to methanol was used as mobile phase, and the phenolic acids gradually eluted faster when the proportion of acetonitrile in the mobile phase was increased [17]. The extraction conditions of procyanidins (PC) from the Chinese hawthorn (*C. pinnatifida* Bge. var. *major* N.E.Br.) fruits were optimized by response surface methodology (RSM). Results showed that 93.4 ± 0.21% of the procyanidins could be recovered. The crude extract was then purified by using a LSA-10 resin column, which showed excellent adsorption and desorption properties for PC purification. A fraction with PC content above 83.2% and mainly consisting of EC, a singly-charged dimer and trimer as identified by HPLC/MS, was obtained by isolation on LSA-10 resin. Liu *et al*. [7] investigated microwave-assisted extraction of polyphenols from *C. pinnatifida* Bge. The best extraction parameters were obtained using RSM. Under this condition, an extraction yield of 23.5% was obtained and the percentage of phenolic compounds in the extract reached 41.2%. Wu *et al*. [49] utilized a heat-reflux extraction (HRE) method for extraction of rutin and quercetin from Chinese hawthorn berries. This optimized method achieved the highest efficiency in the shortest extraction time with the least solvent consumption.

Temperature was found to markedly influence the stability of polyphenolics in Chinese hawthorn berries. Low temperatures are recommended for storage of fruit. Chang *et al*. [51] studied the stability of five dominant phenolic compounds: epicatechin, procyanidin B2 (PC B2), chlorogenic acid (ChA), hyperoside (HP) and isoquercetin (IQ) in hawthorn fruits and a canned hawthorn drink during six months of storage in the dark and at three different temperatures (4, 23 and 40 °C). The results showed that the studied phenolics were stable at 4 °C and relatively unstable at 23 and 40 °C in the fruit and drink. At 23 °C they observed a significant degradation of EC and PC B2 around 50% and 30% after six months of storage. The most significant degradation in studied components was found at 40 °C.

### 2.6. Relationship between Individual Polyphenolic Compounds

Correlation analysis of the levels of the seven compounds epicatechin, procyanidin B2, procyanidin B5, procyanidin C1, hyperoside, isoquercitrin and chlorogenic acid in fruits of 37 cultivars of *C. pinnatifida* Bge. yielded a strong correlation (*p* < 0.001) between the individual contents of the four procyanidins and total procyanidin contents (*r* = 0.7413–0.9898), and between flavonoids and the chlorogenic acid (*r* = 0.5383–0.9212) [25]. Moreover, Person’s correlation coefficient analyses were used to investigate the correlation relationship between the content of individual polyphenols in Chinese hawthorn fruits. Cui *et al*. [10] determined a positive correlation between the contents of hyperoside and isoquercitrin (*r* = 0.49, *p* < 0.01), and a significant positive correlation was found between the content of epicatechin and the levels of practically all procyanidins, except the PA dimer hexoside. A positive correlation also exists between the contents of PA B2 and PA trimer II. A weak negative corelation between total flavonols and total PAs was also observed (*r* = −0.16).

## 3. Biological Activity of Polyphenols in Chinese Hawthorn Fruit

Antioxidant activity of hawthorn berries rich in phenolic compounds have been widely used as both a medicinal and food raw material in China and Europe [3,8].

### 3.1. Antioxidant Activity of Chinese Hawthorn Fruit

Antioxidant activity refers to the ability of antioxidants to quench or scavenge free radicals [52,53,54,55]. Recent studies have shown that the extracts of *C. pinnatifida* Bge. have the capacity to quench free radicals and inhibit the oxidation of low density lipoprotein (LDL) in both cells and cell-free systems [56,57,58]. Fruit constituents found to be responsible for free radical scavenging activity are epicatechin, hyperoside and chlorogenic acid, and these compounds are considered to be the best antilipoperoxidants [10]. The antioxidant profile of hawthorn fruits was also studied and it was found that it could be represented by eight pure compounds isolated by chromatography, namely ursolic acid, hyperoside, isoquercetin, epicatechin, chlorogenic acid, quercetin, rutin and protocatechuic acid [59,60]. All these compounds, except ursolic acid, protected human low-density lipoprotein (LDL) from Cu^2+^ mediated LDL oxidation. They were also effective in preventing the peroxy free radical-induced oxidation of α-tocopherol in human LDL. The inhibitory effect of these compounds on oxidation of LDL and α-tocopherol was dose-dependent at concentration 5–40 µM DM. Chu *et al*. [56] studied a hot water extract of dried fruit of *Crateagus pinnatifida* and found its capacity of quenching 1,1-diphenyl-2-picrazyl radicals to be E_C_ = 0.118 mg/mL. Flavonoids (6.9%), procyanidins (2.2%), catechin (0.5%) and epicatechin (0.2%) are responsible for this high antioxidant capacity. A hot water extract also reduced LDL oxidation in a Cu^2+^-induced cell-free system as well as in sodium nitroprusside (SNP)-treated RAW 264.7 (a mouse leukaemic monocyte macrophage cell line) [56]. The ethyl acetate fraction of the ethanol extract of *C. pinnatifida* fruits also protected LDL from Cu^2+^-induced oxidation *in vitro*. Studies by Zhang *et al*. showed that the Chinese hawthorn fruit is an excellent source of constituents with antioxidant properties purified from ethyl acetate extract reduced production of thiobarbituric acid-reactive substances (TBARS) in Cu^2+^-induced LDL oxidation and inhibited peroxy radical-induced oxidation of α-tocopherol in LDL and in AAPH assay [27]. In this work quercetin, isoquecetin and hyperoside showed stronger effects than other phenolic compounds.

Biological studies showed that the extract from hawthorn (*C. pinnatifida* Bge.) possessed a strong inhibitory effect against DPPH, hydroxyl radicals and lipid peroxidation, as well as strong reducing power [7]. Bahorn *et al*. [59] examined the antioxidant activity of hawthorn extract and found out that it could also scavenge hydrogen peroxide and superoxide species.

Procyanidins also play a very important role in antioxidant activity [10]. The procyanidin fraction from fruits of *C. pinnatifida* var. *major* scavenged superoxide and hydroxyl radicals and inhibited lipid peroxidation *in vitro* [61]. The antioxidant activity of hawthorn procyanidins was tested *in vitro* with different systems. In solution systems, the ^.^OH and O_2_^−^ scavenging ability of hawthorn polyphenolic compounds was higher than that of vitamin C. In a liposome peroxidation system, hawthorn polyphenolic compouds exhibited much higher antioxidant activity than vitamin E. In addition, hawthorn polyphenolic compounds (0.02% w/v) efficiently inhibit lipid peroxidation during enzymatic hydrolysis of porcine meat at 50 °C for 24 h [7]. Three oligomeric procyanidins isolated from *C. pinnatifida* leaves and fruit exhibited collagenase inhibitory activity (IC_50_) at concentrations of less than 1 µM [62].

Crude extracts of *C. pinnatifida* Bge. fruits were stronger than the purified one in their antioxidant activity, especially in the solution system. The ethyl acetate extract fraction consisting of procyanidins with DP 1–5 (197 g/kg), chlorogenic acid (12 g/kg DM) and flavonoids (5 g/kg DM) inhibited the activity of prolyl endopeptidase and showed scavenging activities on superoxide and hydroxyl radicals [10]. Another chemoprotective effect of phenolic compounds is the inhibition of oxidative enzymes like tyrosinase and lipoxygenase.

Li *et al*. compared the antioxidant, anti-α-glucosidase and anti-inflammatory activities of *Crateagus pinnatifida* Bunge var. *typica* Schneider (CBS) and *C. pinnatifida Bunge* (CB) [58]. The CB showed significantly higher antioxidant activity than CBS extract in several antioxidant property tests. They also measured the inhibitory activity on lipopolysaccharide (LPS)-induced nitric oxide (NO), production and pro-inflammatory iNOS and COX-2-mRNA levels. In this way CB is more suitable for further anti-oxidative research, however, the CBS is more effective for prevention of inflammatory related diseases. Among fruit types of less common fruit species—honeyberry (*Lonicera kamtschatica* Sevast. Pojark), Saskatoon berry (*Amelanchier alnifolia* Nutt). and Chinese hawthorn (*Crateagus pinnatifida* Bge.), Hawhorn fruit showed the highest antioxidant capacity by both the TAC and DPPH assay methods [14].

### 3.2. Biological Activity of Fruits

Generally, fruits of hawthorn (*Crataegus* sp.) are used both in traditional and folk medicine to improve digestion, avoid food retention, promote blood circulation and resolve blood stasis [27,63]. Traditionally, the fruits are used for their astringent properties in heavy menstrual bleeding and in diarrhoea. Berries act as diuretics and can be used to treat kidney problems and dropsy [64]. At the same time, they show numerous mild, but well documented pharmacological activities [27]. Fruits of the Chinese hawthorn (*C. pinnatifida* Bge.) berries displaying a high level of flavonoids and procyanidins [10,17,22] are considered the key bioactive of the hawthorn, offering antioxidative, free radical scavenging, and hypolipidemic effects [18,64,65,66,67]. The pharmacological effects of *Crataegus* spp. have mainly been attributed to their individual polyphenolic compounds such as oligomeric procyanidins (OPCs), abundant in hawthorn [7].

#### 3.2.1. Hypolipidaemic Effects

During the last few decades Chinese hawthorn fruit has received much attention because of its potential to reduce serum total cholesterol, low-density lipoprotein cholesterol (LDL-C) and triacylglycerols (TAG) level in animal models [13,27,68,69,70,71,72,73] as well as studies in hyperlipidemic humans [74,75].

After 12 weeks, serum total cholesterol (TC) and triglyceride (TG) were 23.4 and 22.2% lower, respectively, in the Chinese hawthorn fruit group of New Zealand white rabbits on a high cholesterol diet compared with the control group (*p* < 0.05) [13]. In a similar experiment with hamster serum total cholesterol (TC) and triacylglycerols (TG) were decreased by 10 and 13%, respectively, in the Chinese hawthorn group as compared with a control group fed with a semisynthetic diet containing 0.1% cholesterol (*p* < 0.05). Polyphenolic extract of hawthorn fruit lowered serum total cholesterol (TC) and low-density lipoprotein cholesterol (LDL-C) and inhibited the accumulation of hepatic TC and triglyceride (TG), improved antioxidant status in cholesterol—enriched diet (CED) for 12 wkd in male ICR mice. Also hepatic histopathological examinations showed markedly decreased fatty deposits in the liver of mice treated with extract [76].

Results of comparative studies by Min *et al*. [77] of the anti-hyperlipidemic effect of red ginseng (root of *Panax ginseng* C.A. Meyer) and Crateagii fructus (CF, the fruit of *C. pinnatifida* BGE) are also very interesting. Treatment of RG and CIF significantly reduced blood triglyceride (TG) and total cholesterol (TC) levels in Triton WR-1339-induced hyperlipidemic mice and serum TG levels, decreased blood HDL cholesterol in corn oil-induced hyperlipidemic mice.

Rajendran *et al*. showed that the hypolipidemic effects could be associated with flavonoid and triterpene saponins in fruits as well [69]. In addition, Ye *et al*. isolated 3-hydroxy-3-methylglutaryl coenzyme A reductase inhibitors from hawthorn fruit (*C. pinnatifida*) and evaluated them for their antihyperlipidemic effect in mice subjected to a high-fat diet [78]. They discovered that the inhibitory rate of a mixture of compounds (quercetin, hyperoside, rutin and chlorogenic acid) was up to 79.5%, much higher than that of the single compounds. *In vivo* results also revealed that the mixture had a more significant lipid-lowering efficiency than monomers. Structure-activity relationships revealed the inhibitory activity and lowering-lipid ability of quercetin, hyperoside and rutin decreased with increasing glycoside numbers.

Recent studies have been aimed at clarifying the bioactive substances in Chinese hawthorn fruit responsible for this action and the underlying mechanism. In the study reported by Rajendran *et al*. [69], supplementation of 0.5 mL alcoholic extract of *C. laevigata per* 100 g body weight *per* day for 6 weeks was associated with a significant increase of hepatic LDL-receptor activity, resulting in greater influx of plasma cholesterol into the liver. Extract also prevented the accumulation of cholesterol in the liver by enhancing cholesterol degradation to bile acids and by simultaneously supressing cholesterol biosynthesis. This probable mechanism was also supported by a study by Ho *et al*. [79], who investigated the effect of hawthorn fruit extract on HepG2 cells and demonstrated a significantly increased LDL-receptor activity. The reduction in serum total cholesterol by dietary hawthorn fruit extract is a complex process that involves multifaceted interactions of cholesterol metabolism. This process involves a greater extraction of bile acids mediated by up-regulation of hepatic cholesterol 7α-hydroxylase activity, and an inhibition of cholesterol absorption mediated by down-regulation of intestinal acyl CoA, cholesterol acyltransferase (ACAT) activity. Therefore, another possible mechanism for the hypocholesterolemic activity of hawthorn fruit could be either inhibition of cholesterol and bile acids absorption or increased excretion of neutral and acidic sterols. Intestinal ACAT play a key role in absorption of cholesterol by esterification of cholesterol before absorption. Therefore, Zhang *et al*. determined whether hawthorn fruit reduced the absorption of cholesterol and investigated the ACAT activity in the intestine of rabbits [13]. Studies proved that non-aqueous ethanolic extract of hawthorn (*C. pinnatifida*) could suppress intestinal acyl-Coa:cholesterol acyltransferase (ACAT) activity in rabbits. The hypolipidaemic effects of the hawthorn phenolic extract were associated with the activation of peroxisome proliferator-activated receptor α (PPAR α) in the adipose tissue of hamsters and were reversed by treatment with MK 886, a PPAR α antagonist [80]. The hypolipidaemic effects of phenolic extract of hawthorn can be partially mediated by regulation of the activity of lipoprotein lipase (LPL) in muscle and adipose tissue [81]. The oral intake of increase of PPAR alpha expression to facilitate β-oxidation related enzymes in liver for lipid degradation and blood lipid decreases [82].

#### 3.2.2. Effects on Cardiovascular Diseases and the Vascular System

Aqueous alcohol extract of hawthorn fruits, especially standardised extracts of the European hawthorn (*C. monogyna* or *C. laevigata*), are used as dietary supplements and herbal medicines for treating heart failure and mild forms of arrhythmia [82,83,84] and also to treat angina pectoris [85] and all specimens of the *Crataegus* sp. Genus, including *C. pinnatifida*, are recommended for treatment of myocardial weakness, paroxysmal tachycardia, hypertension and arteriosclerosis [24]. Many recent clinical trials show benefit concerning the objective signs and subjective symptoms of New York Heart Association (NYHA) stages I–II congestive heart failure [12,83]. Therefore, Weihmayer *et al*. recommended *Crateagus* spp. as an effective and safe therapeutic alternative for this indication [1]. Hwang *et al*. found out that hawthorn treatment modifies left ventricular remodelling and counteracts myocardial dysfunction in early pressure overload-induced cardiac hypertrophy [86].

Consumption of hawthorn fruit is also associated with long-term benefits to the cardiovascular system, partially due to its effect on serum cholesterol [11,63]. This mechanism might also involve the direct protection to human LDL from oxidation or indirect protection via maintaining the concentration of α-tocopherol in human LDL [27]. In High-cholesterol diet (HCD)—fed rats, an increased plasma total cholesterol and LDL-cholesterol with a decreased HDL-cholesterol was observed, and consumption of hawthorn markedly suppressed the elevated total cholesterol and LDL-lipoprotein levels plus an increased HDL cholesterol level. The blunted acetylcholine-induced, endothelium-dependent relaxation of isolated aortas of HCD-fed rats was improved by hawthorn. In addition, hawthorn fruit significantly inhibited tromboxane A 2 biosynthesis and platelet adhesion, thus reducing the formation of atheroma and thrombosis [87].

Some *in vitro* studies have shown that hawthorn extracts exert endothelium-dependent vasorelaxation effects [65,86]. It is also interesting that the procyanidin fraction isolated from a standard hawthorn extract presented remarkable vasorelaxation effects, but the flavonoid fraction did not show any such observable effect [86,87]. Oligomeric procyanidins have been reported to relax endothelium blood vessels at remarkably low concentrations of hawthorn ethanol fruit extract as a special inhibitor of endothelin-1-release displayed hypotensive effect [8].

Many studies have demonstrated the beneficial effects of extracts of hawthorn berries on the blood circulation system [88]. Hawthorn fruit extract also improved coronary circulation [12,89]. An increase of coronary flow caused by the *O*-glycosides luteolin-7-glucoside (186%), hyperoside (66%) and rutin (66%), as well as an increase of the relaxation velocity by luteolin-7-glucoside (104%), hyperoside (62%) and rutin (73%) were markedly effected of flavonoids from *Crateagus s*pecies observed at a maximum concentration of 0.5 mmol/L [31]. Similar but less intensive effect was found with the *C*-glycosides of vitexin, vitexin rhamnoside and monoacetylvitex rhamnoside. The possible mechanism of cardiac action of flavonoids extracted from hawthorn berries suggest the inhibition of 3'-5'-cyclic adenosine monophosphate phosphodiesterase [89], while another study also showed that the consumption of flavonoid antioxidants is inversely correlated with risk of coronary heart disease [13].

Current research to date suggests that hawthorn may potentially represent a safe, effective, nontoxic agent in the treatment of cardiovascular disease (CVD) and ischemic heart disease (IHD) [2]. Because a patient with cardiac illness taking digoxin may also take hawthorn, Dasgupta *et al*. investigated potential interference of hawthorn in serum digoxin measurements using immunoassays as well as pharmacodynamic interaction between hawthorn and digoxin [90]. They found out interference of hawthorn with a digoxin immunoassay and pharmacodynamic interaction with digoxin, thus reaching the conclusion that a patient receiving digoxin should avoid hawthorn.

Currently, hawthorn leaves, flowers and both green (unripe) and red (ripe) berries are used to make herbal preparations to treat patients with cardiovascular disease [62]. In China Myakuru-herbal medicine (MR) made up of *Panex notoginseng*, *Ginko biloba* and *C. pinnatifida* Bge is widely used. Iwaoka *et al*. showed the preventive effects of the medicine in hypertensive rats, whereby systolic blood pressure in MR-treated rats was significantly decreased, cerebral blood flow in the MR treated group was significantly higher than in control group [91]. Among therapeutic food supplements, hawthorn fruit (*Shan Zha*) is known in Traditional Chinese Medicine (TCM) for its effects on reducing food stagnancy and blood stasis, improving blood circulation and is used to treat hypertension [92]. As a herbal medicine, *Shan-Zha* fruit extract showed merit in improving obesity and hyperlipidemia. Studies with fatty rats and hamsters show that after administration of *Shan-Zha* significant decreases of body weight, plasma TG, TC (total cholesterol) and FFA (free fatty acids), liver TG, TC and FFA, plasma alanine transferase (ALT) and aspartic aminotransferase (AST) compared with control group occurred [76,93]. *Shan Zan* inhibited the fat droplet accumulation in adipocytes. The clinical efficacy of the Chinese therapeutic food (specifically hawthorn fruit and Chinese kiwifruit-extract compound) on dyslipidemia was evaluated in a placebo-controlled, double blind, paired clinical trial conducted in Melbourne, Australia. The results indicated that a four-week intake of the compound increased the serum HDL-c levels by 5% (*p* = 0*.*026) and decreased the ratio of TC/HDL-c and LDL-c/HDL-c (*p* = 0*.*012 and *p* = 0*.*044, resp.). The intake of hawthorn fruit and Chinese kiwifruit extract compoundd may increase the serum levels of HDL-c and decrease the ratios of TC/HDL-c and LDL-c/HDL-c, therefore, may reduce the risk of cardiovascular disease [94].

#### 3.2.3. Other Activities

Kao *et al*. demonstrated that the flavonoid contents of dried fruits of C. *pinnatifida* present *in vitro* and *in vivo* anti-inflammatory potential, and may play a role in hepatoprotection [57]. The preliminary investigation showed that *Crateagus* flavonoids (CF-Fs) (0.25–0.75 mg/mL) decreased the release of PGE_2_ and nitric oxide (NO) as induced by lipoplysaccharide (LIPS and endotoxin) in macrophage RAW 264.7 cells. In addition, oral administration of *Crataegus* spp. fruit extract caused a dose-dependent antiinflammatory effect in a model of carrageenan-induced rat paw edema.

Antimicrobial testing of the *Crataegus* sp. extract revealed its moderate bactericidal activity, especially against G-positive bacteria *Micrococcus flavus*, *Bacilus subtilis* and *Lysteria monocytogenes* with no effect on *Candida albicans* [19].

Gastroprotective activity of *Crataegus* fruit extract was investigated using an ethanol—induced acute stress ulcer in rats model, with rantidine as reference drug. Hawthorn extract produced dose-dependent gastroprotective activity (3.8 ± 2.1, 1.9 ± 1.7 and 0.7 ± 0.5 for doses 50,100 and 200 mg/kg, respectively). Consumption of hawthorn (*C. pinnatifida*) altered the digestive enzymes of the stomach and cholesterol metabolism of the liver [19,68].

Procyanidins show cytotoxic potential on SK-OV-3 cancer cell lines. Vitexin-2''-*O*-rhamnoside (VOR) is the polyphenolic compound presented in the leaves of *C. pinnatifida* Bge.var. *major* and in minor proportion in fruit. VOR plays an important role in preventing human pathologies related to oxidative stress [48]. Oligomeric procyanidins isolated from extract of *C. pinnatifida* exhibited collagenase inhibitory activity (IC_50_) at a concentration of less than 1 mµ/M [62]. Kao *et al*. [9] proved chemopreventive role of *C. pinnatifida* fruit. They use JB6 mouse epidermal cell model to investigate the molecular events specific to tumour promotion. The achieved results of experiment demonstrated that polyphenolic fraction of dried fruit of *C. pinnatifida* inhibits TPA (12-0-tetradecanoylphorbol-13-acetate) induced tumour transformation by blocking the AP-1 and NF- B signals, protein expression of COX-2/iNOS, generation of H_2_O_2_, activation of MPO and tumour promotion by decreasing inflammation and oxidative stress [9].

The oligomeric procyanidins fraction of *C. pinnatifida* fruit showed notable growth inhibitory activity against chloroquine-sensitive strains of *P. falciparum* with IC_50_ values of 2.7 μM (SI values of >55.5). This is the first report on the antiplasmodial activity of these oligomeric procyanidins from Chinese hawthorn fruits [95].

## 4. Conclusions

There has been an increasing interest in investigating polyphenols from lesser known fruit species because of their potential health benefits in the prevention of chronic diseases. Polyphenols represent the predominant group of biologically active substances in the Chinese hawthorn (*Crataegus pinnatifida* Bge.) fruit, responsible for its hypolipidemic effects and reduction of the risk of cardiovascular diseases (myocardial weakness, paroxysmal tachycardia, hypertension, arteriosclerosis, angina pectoris, congestive heart failure) and cancer. Polyphenols responsible for free radical scavenging activity are epicatechin, hyperoside and chlorogenic acid, and these compounds are considered to be the best antilipoperoxidants. Procyanidins, especially procyanidin B2, procyanidin B5 and procyanidin C1 also play a very important role in its antioxidant activity, as the procyanidin fraction from fruits of *C. pinnatifida* var. *major* scavenges superoxide and hydroxyl radicals and inhibits lipid peroxidation *in vitro*. The polyphenolic content of Chinese hawthorn fruit is dependent on the cultivars, the locality of cultivation, the stage of maturity, the conditions of extract preparation and the method of chemical determination of polyphenols.
